# Enhancing, Understanding and Adoption of the Contributor Roles Taxonomy (CRediT)

**DOI:** 10.1002/leap.2048

**Published:** 2026-03-19

**Authors:** Mohammad Hosseini, Simon Kerridge, Liz Allen, Veronique Kiermer, Kristi Holmes

**Affiliations:** 1 Department of Preventive Medicine, Northwestern University Feinberg School of Medicine, Chicago, Illinois, USA; 2 Galter Health Sciences Library and Learning Center, Northwestern University Feinberg School of Medicine, Chicago, Illinois, USA; 3 Research and Innovation Services, University of Kent, Kent, UK; 4 Research Consulting, Nottingham, UK; 5 Policy Institute, King’s College London, London, UK; 6 Public Library of Science, San Francisco, California, USA

**Keywords:** authorship, contributor roles, CRediT, publication ethics, research integrity

## 1 | Introduction

Introduced for widespread use in 2015, the Contributor Roles Taxonomy (CRediT) offers 14 standard roles to classify scholarly contributions to research outputs (Allen et al. 2014). In 2022, CRediT was formalized as a standard by the American National Standards Institute (ANSI) and the National Information Standards Organisation (NISO), facilitating a sustained support system.

Thus far, several key academic workflows, including journal submission and review systems (e.g., Editorial Manager, PubSweet, ScholarOne, ReView, and the Open Journals System) have incorporated CRediT, resulting in its adoption by hundreds of journals across many major publishers including PLOS, SAGE, Springer Nature, Wiley, Frontiers, and Elsevier. That said, a recent retrospective study showed that CRediT has been inconsistently integrated among publishers, research domains and countries (Allen et al. 2025a). For example, while PLOS has fully implemented CRediT and mandates it across its portfolio, other publishers started much smaller and without a mandate. Elsevier, MDPI, Springer Nature, and Frontiers are the four publishers with the highest number of publications that include CRediT roles. In terms of research domains, publications in Engineering, Biomedical and Clinical Sciences, Biological Science and Chemical Sciences have the highest rate of CRediT inclusion. When considering the country of the corresponding author, the largest number of articles with CRediT information comes from China, followed by those based in the U.S., India, and the U.K. According to this study, in 2024, 22.5% (*n* = 848,841) of original research articles, preprints, and conference papers with available full text indexed on the Dimensions platform employed the CRediT taxonomy to specify author contributions.

Further integration of CRediT in the scholarly landscape faces both technical and social challenges. Examples of technical challenges include difficulties of incorporating the taxonomy in legacy submission and hosting platforms that were not originally designed to support structured contributor role metadata. Moreover, the lack of standardised implementation of CRediT across publishers and platforms leads to inconsistent metadata. For example, while PLOS has integrated CRediT into its submission workflow, many other publishers and journals collect CRediT information only as narrative declarations at the end of a manuscript, meaning that these contributions are not carried forward as structured metadata. There is also limited interoperability between manuscript submission systems, publishing platforms, and downstream metadata services (e.g., indexing and discovery systems), which makes tallying contributions complicated. Social challenges include training researchers on how to use CRediT and for what purpose (i.e., “CRediT is NOT intended to define what constitutes authorship – but instead to describe the specific contributions of authors and other contributors that result in scholarly output”); how to accurately assign contributor roles and develop shared norms around appropriate role attribution in the presence of hundreds of authors; preparing editors to monitor usage, resolve disputes, and address potential misuse of roles; and creating institutional practices that accommodate the use of contributor roles in evaluations.

In 2020, the CRediT Standing Committee (CSC) was established to help to manage and govern the CRediT and ensure its inclusive and transparent evolution. One of the goals of the CSC is to enhance awareness and understanding of CRediT and the value it can add to scholarly activities, thereby supporting its further adoption across the research community.

## 2 | New Resources for the Community

The CSC has developed several initiatives as part of a broader program of activity to support uptake, create awareness and receive community feedback. To directly address some of the challenges identified by the CSC and the broader scholarly community, we are excited to share two new resources.

### 2.1 | Example Research Tasks That Could Be Described Using CRediT Roles

In September 2022, the CSC organised two online workshops consisting of 14 breakout sessions (NISO 2022). These workshops were open to anyone interested in joining and were promoted through NISO website and social media networks. Role experts (familiar with CRediT and bringing specific experience and expertise in one of the roles) facilitated open discussions among participants. Moderators collected comments and suggestions about existing use cases and the current challenges that may occur when assigning specific roles to individuals. In the following months, members of the CSC held several meetings to assess the offered suggestions and recommendations. One of these suggestions was to develop examples that show which specific research tasks could be described by each CRediT role. The CSC agreed that this is a helpful idea and supported the creation of example tasks. Using the collected information from the workshop, the CSC drafted an initial list and after numerous iterations and discussion sessions, finalised the list of examples (Hosseini 2025). While the example tasks are neither exclusive nor prescriptive, they provide illustrative use cases that can help individuals to determine which CRediT role might best describe certain contributions. For each role, several example tasks have been suggested (Table 1).

These examples have been added to the specific page for each role on the CRediT website, hosted by NISO (see for instance, the page for the role of Conceptualization here: https://credit.niso.org/contributor-roles/conceptualization/), and uploaded to Zenodo as a citable item (Hosseini et al. 2026). Examples can be freely used by journals, universities, libraries and others that are interested in promoting responsible use of CRediT and more accurately representing the range of scholarly contributions. The CSC believes that these examples facilitate a better understanding of roles for researchers, administrators, and community members, and can support them to acknowledge and value the diversity of contributions required in research. Indeed, by making it easier to comprehend and relate to roles, these examples provide a richer, more nuanced understanding of various ways individuals might contribute to the research process.

### 2.2 | Unique Badges for Enhancing Clarity of the CRediT Standard

As we build a standard for describing contributions and promote the implementation of CRediT, new strategies are needed to ensure that CRediT is used correctly and as intended. The CSC has been exploring how to use simple badges for each role to give them a clear identity. These badges could be used in various interfaces such as journal websites or editorial software. Furthermore, the use of badges can help the user be sure that the original ANSI standard CRediT has been used, potentially boost recognition of CRediT, and further increase its adoption.

When linked with ORCIDs (Open Researcher and Contributor IDs), CRediT role badges could be part of a streamlined process to visually represent skills and tally an individual’s contributions to research. As part of our roadmap, the CSC intends to further explore the value of these badges with potential users and learn about the practical challenges associated with developing and integrating them into relevant platforms and systems (e.g., manuscript submission systems, scholarly and bibliographic indexes).

## 3 | The Road Ahead

### 3.1 | Evolution of CRediT Roles

We are still using and promoting the CRediT 1.0, which was released in 2015. Since then, research and scholarly contributions have evolved in numerous ways. Take for instance, the rapid growth of data-intensive science and an increasing footprint of artificial intelligence in research, increasing recognition of community and teaching activities as part of formal research projects, and continual evolution of techniques and tools. Several studies have proposed additional roles to be included in the CRediT taxonomy or suggested revising the definition of existing roles (Hosseini et al. 2024). We are aware of the need to update CRediT roles, and some of us have also been involved in proposing roadmaps for how this process could look like (Hosseini et al. 2023). However, on the one hand, the diversity of proposed changes illustrates that researchers interpret the research workflow from discipline- and role-specific perspectives, making it unlikely that a single taxonomy could ever accommodate all views. On the other hand, expanding the taxonomy to include many additional roles or updating it too frequently would reduce its cross-disciplinary applicability and generalizability and undermine its usability. More importantly, undertaking such revisions requires substantial coordination, governance, and resources that are currently unavailable (Allen et al. 2025b).

### 3.2 | Publishers’ and Crossref Integration of CRediT

Many publishers already capture and include CRediT information in their article metadata. However, because Crossref has not previously aggregated these data, they have not been consistently visible in downstream systems that rely on Crossref records. In July 2024, Crossref announced plans to include CRediT metadata within their workflows (Schema Development Plans 2024). Crossref’s adoption will therefore enable the centralised aggregation and broader dissemination of publisher-supplied CRediT metadata (e.g., to ORCID), facilitating improved documentation and tracking of author contributions, greater transparency, and more consistent attribution in scholarly publishing. This information would be incorporated into article metadata alongside other structured elements, such as author, institutional, and funding metadata. Once fully integrated, Crossref’s infrastructure will allow CRediT roles to be more systematically surfaced and reused across the research ecosystem, supporting collaboration assessment and enabling institutions and funders to evaluate research contributions more accurately over time.

### 3.3 | The CRediT Standing Committee Will Continue to Support Users

The CSC is keen to develop practical approaches that help support the uptake of CRediT and make it easy for different members of the community to understand how it can be used in practice. As such, the CSC supports metaresearch activities that enhance the research community’s understanding of CRediT implementation and create awareness about challenges and is willing to provide feedback to such efforts or share them with the larger community when they are deemed generalizable and helpful. Under *Resources about CRediT* (https://credit.niso.org/of-interest/), the NISO CRediT page offers a list of articles and resources that can support researchers, editors, and administrators interested in adopting CRediT. As mentioned earlier, despite the increasing uptake and CSC’s ongoing efforts, challenges associated with CRediT adoption within a diversity of scholarly workflows and domains persist. In an attempt to collectively address these issues, we invite all members of the scholarly community to register to join our soon-to-be-set-up CRediT Community Interest Group and participate in our commitment to responsible and accurate attributions. There will be reflection points and opportunities for the user interest group to get involved and provide input. Working together we can contribute to advancing uptake and responsible use of CRediT.

## Figures and Tables

**TABLE 1 |:** CRediT roles and example research tasks that could be attributed to them.

Roles and their definitions	Example tasks
*Conceptualization:* ideas; formulation or evolution of overarching research goals and aims.	Identifying issues, questions or problems that warrant research. Developing research questions and hypotheses. Developing research frameworks, tools or experimental paradigms. Refining and adapting overarching research goals and aims.
*Data curation*: management activities to annotate (produce metadata), scrub data and maintain research data (including software code, where it is necessary for interpreting the data itself) for initial use and later re-use.	Conducting tasks like data processing, cleaning, cataloguing, annotating, archiving modelling, and retention. Integrating and aggregating data in diverse formats and from diverse sources. Managing and updating data descriptions and metadata, including maintaining version control and associated documentation. Developing or implementing data preservation strategies to ensure data remains findable, accessible, interoperable and reusable.
*Formal analysis:* application of statistical, mathematical, computational or other formal techniques to analyse or synthesise study data.	Uncovering patterns and identifying relationships between variables and quantitative or qualitative datasets. Performing statistical tests to compare different groups within a study or evaluate change. Applying AI and machine learning models to predict outcomes. Developing computational simulations to model complex systems or phenomena.
*Funding acquisition:* acquisition of the financial support for the project leading to this publication.	Identifying suitable funding sources, assessing eligibility and communicating requirements with the team members. Developing grant proposals and coordinating the submission process. Developing budgets and allocating funds to match project scope and funder expectations.
*Investigation:* conducting a research and investigation process, specifically performing the experiments, or data/evidence collection.	Following or modifying methods to collect or generate data through, for quantitative and/or qualitative research approaches. Testing research hypotheses and documenting the research process. Searching and reviewing the literature, samples, data and other evidence. Reporting findings for further discussion, analysis, and exchange of ideas.
*Methodology:* development or design of methodology; creation of models.	Developing quantitative and/or qualitative methodologies and frameworks. Defining search strategies and determining criteria for systematic literature reviews. Determining study design such as participant selection, materials, settings, data characteristics, data collection, measurement, and analysis techniques.
*Project administration:* management and coordination responsibility for the research activity planning and execution.	Monitoring and reporting progress, timelines, budgets, and compliance with ethical, governance, legal, health, safety, and other relevant standards. Recruiting participants needed for the research method (e.g., for interviews, focus groups, surveys, fieldwork, clinical trials). Organising logistics for expeditions, fieldwork, equipment setup, and space allocation that support research operations. Managing correspondence with team members, journal editors, and various institutional departments.
*Resources:* provision of study materials, reagents, materials, patients, laboratory samples, animals, instrumentation, computing resources, or other analysis tools.	Preparing, transporting or managing access to samples, artefacts, tools, equipment, documents, archives and digital/physical infrastructure. Inventory management, safekeeping of samples and providing reports on availability and state of resources. Calibrating and maintaining instruments and equipment. Coordinating data storage solutions and computational resources.
*Software*: programming, software development; designing computer programs; implementation of the computer code and supporting algorithms; testing of existing code components.	Designing, developing, testing, debugging, implementing, documenting, sharing and maintaining code. Developing, maintaining, managing and optimising digital infrastructure, libraries, and databases. Conducting data extraction, data mining, and parsing content for qualitative or quantitative data collection and analysis. Ensuring interoperability, functionality and scalability of code, databases, systems or platforms across different environments.
*Supervision:* oversight and leadership responsibility for the research activity planning and execution, including mentorship external to the core team.	Overseeing researchers and other team members by setting milestones, tracking progress, ensuring quality of deliverables and promoting adherence to ethics and integrity norms. Teaching, training, moderating and providing personal or professional advice to team members. Guiding teams in refining methods, interpreting results and addressing interpersonal challenges. Collecting, logging and reporting individual contributions to research.
*Validation:* verification, whether as a part of the activity or separate, of the overall replication/reproducibility of results/experiments and other research outputs.	Ensuring the integrity, rigour and reliability of data, methods, results and resources through reviewing, verification, benchmarking, factchecking and replicating. Conducting pilot tests or preliminary studies to validate data collection instruments and protocols. Appraising studies included in systematic reviews and ensuring compliance with established review standards or reporting frameworks. Testing computational models or simulations against known outcomes for accuracy.
*Visualisation:* preparation, creation and/or presentation of the published work, specifically visualisation/data presentation.	Using data to create charts, graphs or figures. Creating videos and other interactive media for communicating the findings.
*Writing – original draft:* preparation, creation and/or presentation of the published work, specifically writing the initial draft (including substantive translation).	Creating the first and full version of an article. Drafting substantial original text within a section or across sections in an article.
*Writing – review and editing:* preparation, creation and/or presentation of the published work by those from the original research group, specifically critical review, commentary or revision—including pre- or post-publication stages.	Reviewing, copy-editing, refining language and providing comments and suggestions. Revising content based on feedback from internal and external reviewers. Providing review input of figures, tables, and supplementary materials.

## Data Availability

Data sharing not applicable to this article as no datasets were generated or analysed during the current study.

## Key Points

- The CRediT Standing Committee has developed examples of research tasks that can be attributed to each CRediT role.
- Further integration of CRediT within scholarly communication will require ongoing attention to technical, social, and financial challenges.
- Crossref’s adoption of CRediT will centralise and disseminate contributor metadata, improving visibility, reuse, and attribution.
